# The Impact of Agroecosystems on Nitrous Acid (HONO) Emissions during Spring and Autumn in the North China Plain

**DOI:** 10.3390/toxics12050331

**Published:** 2024-04-30

**Authors:** Jianhui Zeng, Wanyun Xu, Ye Kuang, Weiqi Xu, Chang Liu, Gen Zhang, Huarong Zhao, Sanxue Ren, Guangsheng Zhou, Xiaobin Xu

**Affiliations:** 1 State Key Laboratory of Severe Weather, Key Laboratory for Atmospheric Chemistry, Institute of Atmospheric Composition, Chinese Academy of Meteorological Sciences, Beijing 100081, China; zjhcuit@163.com (J.Z.); cliu@cma.gov.cn (C.L.); zhanggen@cma.gov.cn (G.Z.); xuxb01@163.com (X.X.); 2 Institute for Environmental and Climate Research, Jinan University, Guangzhou 511443, China; kuangye@jnu.edu.cn; 3 Guangdong-Hongkong-Macau Joint Laboratory of Collaborative Innovation for Environmental Quality, Guangzhou 511443, China; 4 State Key Laboratory of Atmospheric Boundary Layer Physics and Atmospheric Chemistry, Institute of Atmospheric Physics, Chinese Academy of Sciences, Beijing 100029, China; xuweiqi@mail.iap.ac.cn; 5 State Key Laboratory of Severe Weather, Institute of Agricultural Meteorology, Chinese Academy of Meteorological Sciences, Beijing 100081, China; zhr680317@163.com (H.Z.); 656892rzr@163.com (S.R.); zhougs@cma.gov.cn (G.Z.); 6 Hebei Gucheng Agricultural Meteorology National Observation and Research Station, Baoding 072656, China

**Keywords:** HONO, NH_3_, dew, guttation, soil emission, atmospheric oxidation capacity

## Abstract

Solar radiation triggers atmospheric nitrous acid (HONO) photolysis, producing OH radicals, thereby accelerating photochemical reactions, leading to severe secondary pollution formation. Missing daytime sources were detected in the extensive HONO budget studies carried out in the past. In the rural North China Plain, some studies attributed those to soil emissions and more recent studies to dew evaporation. To investigate the contributions of these two processes to HONO temporal variations and unknown production rates in rural areas, HONO and related field observations obtained at the Gucheng Agricultural and Ecological Meteorological Station during spring and autumn were thoroughly analyzed. Morning peaks in HONO frequently occurred simultaneously with those of ammonia (NH_3_) and water vapor both during spring and autumn, which were mostly caused by dew and guttation water evaporation. In spring, the unknown HONO production rate revealed pronounced afternoon peaks exceeding those in the morning. In autumn, however, the afternoon peak was barely detectable compared to the morning peak. The unknown afternoon HONO production rates were attributed to soil emissions due to their good relationship to soil temperatures, while NH_3_ soil emissions were not as distinctive as dew emissions. Overall, the relative daytime contribution of dew emissions was higher during autumn, while soil emissions dominated during spring. Nevertheless, dew emission remained the most dominant contributor to morning time HONO emissions in both seasons, thus being responsible for the initiation of daytime OH radical formation and activation of photochemical reactions, while soil emissions further maintained HONO and associated OH radial formation rates at a high level, especially during spring. Future studies need to thoroughly investigate the influencing factors of dew and soil emissions and establish their relationship to HONO emission rates, form reasonable parameterizations for regional and global models, and improve current underestimations in modeled atmospheric oxidation capacity.

## 1. Introduction

Nitrous acid (HONO) plays a crucial role in atmospheric chemistry as it is one of the most important sources of the most essential oxidant in the troposphere, hydroxyl radicals (OH), whose abundance is a measure of the atmospheric oxidation capacity and self-cleansing ability [1,2,3]. Daytime OH formation initiates the oxidation of inorganic and organic trace gases as well as aerosol compositions, leading to the formation of secondary pollutants such as ozone (O_3_), peroxyacetyl nitrate (PAN), secondary inorganic aerosols (SIAs) and secondary organic aerosols (SOAs), thereby deteriorating regional air quality and threatening human health [4]. HONO has been reported to contribute 30–60% and 60–92% to atmospheric OH levels during summer and winter, respectively [5,6], leading to simultaneously occurring haze and photochemical gaseous air pollution as indicated by high O_3_ or PAN levels [7,8,9]. Therefore, clarifying the sources of HONO is crucial for the understanding of tropospheric photochemistry and for formulating efficient pollution control strategies. Currently, significant progress has been made in the investigation of direct HONO emissions from various combustion sources, soil and microbial processes, surface photolysis of inorganic and organic nitrates, and both homogeneous and heterogeneous formation processes on different surfaces [10,11,12,13,14,15,16,17]. However, key daytime sources are still missing in the HONO budget, despite all the efforts that have been paid to resolve this issue [18,19,20,21].

The North China Plain (NCP) has been facing severe photochemical and particulate pollution during recent decades, which has received widespread public attention [22,23,24]. Despite significant improvements during the last decade, under stringent emission control measures, the NCP remains one of the most polluted regions in the world, with the ongoing aggravation of ozone pollution and increasing importance of secondary aerosol pollution. The NCP is a traditional agricultural region, with a high coverage of farmlands amidst the heavily populated megacities. Under the increasing demand for food, various natural soils have been transformed into agricultural soils [25] and increasing amounts of chemical nitrogen fertilizers have been extensively applied to cultivated lands, resulting in long-term increases in agricultural nitrogen emissions [26,27]. Thus, cultivated areas might make huge contributions to regional HONO pollution and atmospheric oxidation capacity. Current research in China is mostly focused on the heterogeneous transformation of NO_x_ and associated HONO production on various surfaces such as aerosols, urban grime, architectures, and ground surface [28,29,30,31], and have mainly based their HONO budget and mechanism studies on observations in urban environments [32,33,34,35,36], not paying sufficient attention to agricultural regions that surround the heavily populated urban centers.

Agricultural soil emissions have been proposed to be a major source of HONO in rural NCP, particularly following fertilizing events [16]. Denitrification and anaerobic nitrate reduction within soils are significant sources of HONO and ammonia (NH_3_) under relatively dry soil conditions. Biological nitrate reduction in oxygen-limited micro zones, on the other hand, has been suggested to contribute to HONO emissions under high soil humidity conditions [37,38]. Additionally, recent studies proposed that the natural formation and evaporation of dew and guttation water plays an important role as a nighttime sink and daytime source of HONO and NH_3_ [39], influencing their diel cycles in vegetated areas. Although the potential role of dew water as a storage and emission source of HONO has already been proposed many years ago [40,41,42], it still lacks sufficient recognition and awareness [43]. Dew is formed upon water vapor condensation on ground or plant surfaces due to radiative cooling after sunset [44]. Additionally, various types of vegetation undergo guttation processes that allow their roots to absorb soil water and expel them from hydathodes distributed on leaf tips or edges after sunset, which can continue on for several hours after sunrise under favorable soil humidity conditions [45]. In meteorological observations, guttation is often taken as dew fall. In rural areas, the diurnal temperature variation is generally larger than in urban regions and the humidity is typically higher, thus rendering rural regions more prone to dew formation, with an average dew frequency of around 50% throughout the year in rural NCP [46]. Moreover, the amount of dew and guttation water, which was reported to reach 216 and 750 mL m^−2^ during winter and spring, respectively, is higher than that reported for fog water and exceeds the liquid water content of particulate matter by far [13,47], thus acting as a much more efficient nighttime storage for water-soluble trace gases such as HONO. Aside from that, dew and guttation droplets can also serve as a medium for aqueous phase chemical reactions, providing reaction sites for the conversion of NO_2_ to HONO [43]. Under high NH_3_ conditions such as those found in rural NCP, aqueous phase NO_2_ to HONO conversion might even be further promoted by NH_3_ [46,48].

Although the impact of dew and guttation on HONO deposition, emissions and subsequent OH production rates have been studied for the late autumn season, when winter wheat is sown, the impact in springtime which is the most vigorous growth season of wheat and when there is the highest dew water content has not been studied before. The contribution of soil and dew to HONO emissions and the corresponding OH radical formation rates under stronger radiative conditions, the highly distinct pollution and meteorological conditions in spring, have not been yet evaluated and compared to those during the cold seasons. Therefore, this study adopts HONO, NH_3_, and water vapor observational data acquired during late spring and late autumn campaigns in rural NCP to investigate the relative contributions of dew and soil emissions to atmospheric HONO in those two seasons, and their contribution to the atmospheric oxidation capacity and photochemical air pollution formation.

## 2. Materials and Methods

### 2.1. Measurements and Data

The data used in this study were obtained in field observations conducted at the Gucheng (GC) Agricultural and Ecological Meteorological Station (39°09′ N, 115°44′ E), Chinese Academy of Meteorological Sciences. The spring and autumn campaigns were carried out from 3 April to 5 May 2019 and from 22 October to 23 November 2021, respectively. The GC station is located between two major cities, namely Beijing Municipality (100 km to the northeast) and Baoding City, Hebei Province (~40 km to the southwest) in the North China Plain and is surrounded by farmlands, where winter wheat and summer maize are cultivated as is typical for the entire NCP. Experimental farmlands within the GC station are on the same crop rotation, representing well the pollution and environmental conditions of rural areas in southern Hebei Province [46,48,49,50]. In the spring campaign, the winter wheat was jointing and booting, with wheat leaf area density at its maximum. In the autumn experiment, maize was harvested by the end of October, and wheat was sown in early November, which was at its seedling to tillering stage during the observation period.

Various instruments were placed inside an air-conditioned container on the side of the experimental farm field, to continuously measure HONO, related trace gases (e.g., NH_3_, O_3_, NO_x_) and atmospheric photolysis rates (including *J*(HONO), *J*(O^1^D), *J*(NO_2_), etc.). HONO measurements were performed using the LOPAP-03 analyzer (QUMA Elektronik & Analytik GmbH, Wuppertal, Germany), with zero checks every six hours and liquid phase nitrite standard calibrations every 3–5 days. Calibration liquid standard concentrations of 0.008 and 0.02 mg L^−1^ were used during spring 2019 and autumn 2021, respectively, corresponding to gas phase concentrations of ~1.5 and ~2.2 ppb (conversion from liquid to gas phase concentration depends on gas and liquid sample flow rates), respectively. The instrument has a measurement precision of 1%, with HONO detection limits varying from 1.0 to 6.6 ppt and from 5.2 to 9.6 ppt during the two campaigns, respectively. NH_3_ measurements in spring 2019 were obtained with the economic ammonia analyzer Model DLT–100 (Los Gatos Research, San Jose, CA, USA, precision of 0.2 ppb at 100 s sampling frequency), while the NH_3_ in autumn 2021 was measured using the NH_3_ analyzer Model EC9842 (Ecotech, Knoxfield, VIC, Australia, precision of 1 ppb), which were both calibrated before and after the campaigns using a NH_3_/N_2_ (Air Liquide, Houston, TX, USA, traceable to the US National Institute for Standards and Technology) and a NO_x_/N_2_ (Chemical Metrology & Analytical Science Division, National Institute of Metrology, Beijing, China) reference gas mixture, respectively. O_3_ and NO_x_ were measured using the TE 49C O_3_ analyzer and TE 42C NO_x_ analyzer (Thermo Fisher Scientific, Waltham, MA, USA), where O_3_ was multipoint calibrated before each campaign and NO_x_ was weekly span checked and monthly multipoint calibrated using standard reference gas mixtures. All multipoint calibrations revealed good linearity with R^2^ > 0.999. Photolysis rates were measured using an ultra-fast CCD-spectrometer from METCON GmbH (Lippstadt, Germany), with a spectral resolution below 1.8 nm and a spectral range of 280 to 650 nm. Aerosol chemical composition (including ${NO}_{3}^{-}$, ${SO}_{4}^{2-}$, ${NH}_{4}^{+}$ and organic aerosol mass concentrations) was only measured during the autumn campaign using an aerosol mass spectrometer (AMS, Aerodyne Research, Inc., Billerica, MA, USA), with details on its calibration and data processing described in Kuang, et al.’s paper [51]. Aerosol hygroscopicity was measured using a self-assembled humidified nephelometer system, that can derive aerosol liquid water content as well as ambient aerosol surface area density, details on which can be found in Kuang, et al. [52,53]. Since ${NO}_{3}^{-}$ mass concentrations were not monitored during the spring campaign, it was assumed that ${NO}_{3}^{-}$ made up a fraction of 12% of total PM_2.5_ based on reported springtime observations [54], wherein PM_2.5_ mass concentrations were predicted using humidified nephelometer measurements using machine learning methods described in Xue, et al. [55]. Conventional meteorological data such as atmospheric pressure (hPa), air temperature (°C), soil temperature (10 cm depth, °C), relative humidity (RH), wind direction and wind speed were obtained from an automatic weather station within the site yard.

### 2.2. Data Processing and Definitions of Morning Peaks

Since HONO is very easily photolyzed after sunrise, its daytime formation is often overshadowed by its photolysis loss. To better discuss the variations and potential formation or emission of HONO after sunrise, a photolysis correction was applied to observed HONO concentrations using the following equation:

(1) $${HONO}_{c}=\;{HONO}_{p}\times e^{J\left(HONO\right)\times t}\;$$

where ${HONO}_{p}$ represents the concentration of HONO after photolysis, i.e., the observed concentration, ${HONO}_{c}$ the initial concentration before photolysis, *J*(HONO) the photolysis rate of HONO, and t is the duration of sunlight exposure. HONO and NH_3_ both exhibited typical increases after sunrise, reaching a peak between 8:00 and 10:00 local time (LT), defined as the morning peak. Sometimes they would also reach a peak between 12:00 and 14:00 LT in the afternoon, defined as the afternoon peak. The simultaneous growth and common peak of HONO and NH_3_ was defined as the “dual peak” phenomenon.

### 2.3. HONO Budget Analysis

To evaluate the unknown sources and sinks within the HONO budget, a detailed HONO budget analysis was performed considering currently known and parameterizable HONO generation and consumption pathways. This allows us to estimate a net HONO production rate and by subtracting it from the actually observed HONO variation rate, the currently unexplained HONO production/destruction rate can be obtained, which is denoted as $P_{unknown}$. $P_{unknown}$ > 0 indicates the presence of unknown HONO sources, while $P_{unknown}$ < 0 suggests the existence of unaccounted sinks.

Currently known sources of HONO include primary emissions (from vehicles, biomass burning and soil bacterial/microbial activities) and secondary homogeneous and heterogeneous formation pathways. Among the emission sources, the contribution of vehicle emissions to HONO ($P_{Vehicle}$) is typically estimated based on the ratio between vehicle HONO and NO_x_ emissions (*k*) that was obtained during tunnel experiments [56,57]. Tunnel experiments and car exhaust analyses showed that *k* varied within a wide range of 0.18 to 2.1%. Spataro et al. [58] reported a *k* upper limit of 0.65% for Beijing urban regions in 2013, while Zhang et al. [59] adopted *k* = 0.8% for the Gucheng agricultural site (the same site as in this study), which is used in this study. By assuming that all the observed NO_x_ was contributed by vehicle emissions, an upper limit was obtained for vehicle HONO emissions using:

(2) $$\begin{array}{c} P_{Vehicle}=0.008\times \left[{NO}_{x}\right] \end{array}$$

In the sensitivity study, the variation range of *k* (mentioned above) was applied to test the response of calculation results towards uncertainties in *k*.

Biomass burning events were not encountered during our observation periods and were thus not considered in the budget analysis. Soil emissions (*P_soil_*) are typically quantified by HONO flux measurements [16,38,60,61,62,63], based on which parameterizations of soil HONO emission rates have been proposed, mainly as a function of temperature [61]. Based on such parameterizations, soil emissions revealed very limited influences on HONO during the autumn campaign in our previous study [39]. In this study, another approach was adopted to deduce *P_soil_*, which will be discussed in Section 2.3.

HONO can be directly produced via homogeneous gas phase oxidation of NO by OH radicals:

(R1) $$NO+OH\to HONO,k_{1}\;=\;7.2\;\;\times \;\;10^{-12}\;{cm}^{3}\;molec\;s^{-1}$$

While at the same time, it can also be further oxidized by OH radicals into NO_2_:

(R2) $$HONO+OH\to \;{NO}_{2}+\;H_{2}O,k_{2}\;=\;5.0\;\;\times \;\;10^{-12}\;{cm}^{3}\;molec\;s^{-1}$$

Thus, the overall homogeneous HONO production rate (*P_homo_*) can be calculated as:

(3) $$P_{homo}=\left(k_{1}\times \left[NO\right]-k_{2}\times \left[HONO\right]\right)\times \left[OH\right]$$

Since OH was not directly measured in the two observations, a parameterization based on the strong linear relationship between *J*(O^1^D) and [OH] was adopted following parameterizations proposed based on previous OH observational studies carried out during summer and winter in Wangdu, which is another agricultural site only ~70 km away from GC [64,65]:

(4) $$\mathrm{Spring}\;\left[OH\right]=4.5\;\times \;10^{11}\;\times J\left(O^{1}D\right)+10^{6}$$

(5) $$Autumn\;\left[OH\right]=3.6\times 10^{8}\times J\left(O^{1}D\right)+{1.1\times 10}^{5}$$

To evaluate potential uncertainties caused by the parameterization of OH radicals, we multiplied and divided [OH] by a factor of 2 in the sensitivity study.

Aside from homogeneous gas phase reactions, HONO can also be formed through the heterogeneous conversion of NO_2_ on the ground surface ($P_{ground}$) and aerosol surfaces ($P_{aerosol})$, which are parameterized as:

(6) $$P_{ground}=\gamma_{g}\;\;\times \;\frac{\upsilon \left({NO}_{2}\right)}{8}\;\times \;\frac{S}{V}\;\times \;\left[{NO}_{2}\right],$$

(7) $$P_{aerosol}=\;\gamma_{a\;}\;\times \;\frac{SAC\times \;\upsilon \left({NO}_{2}\right)}{4}\;\times \;\left[{NO}_{2}\right],$$

where $\upsilon \left({NO}_{2}\right)$ is the mean molecular speed of NO_2_, *S*/*V* is the ground surface-area-to-volume ratio (m^−1^), which was assumed to be 1/PBLH according to VandenBoer et al. [66], and SAC is the aerosol surface area density at ambient RH conditions derived from the humidified nephelometer measurements [52]. *γ_g_* is the NO_2_ uptake coefficient on the ground surface, which has been determined to range from 2 × 10^−6^ to 1.6 × 10^−5^ based on nocturnal vertical profile measurements during spring in a rural environment [66], with an average of 8 × 10^−6^. *γ_a_* is the uptake coefficient of NO_2_ on the aerosol surface, which was reported to range from 1 × 10^−9^ (mineral dust) to 7.0 × 10^−6^ (liquid organic aerosols) on different kinds of aerosols under steady states in laboratory experiments [67] (except for sea salt aerosols, which exhibited uptake coefficients between 6.0 × 10^−7^ and 3.0 × 10^−4^). Considering that aerosols in GC mainly comprised secondary inorganic aerosols (sulfate, nitrate, and ammonium salts), primary and secondary organic aerosols, and mineral dust [49], an averaged aerosol uptake coefficient of $1.9\times 10^{-6}$ was adopted. To further account for the enhancement of solar radiation on NO_2_ uptake, as proposed by previous laboratory and field studies [68,69,70], a weighting factor was constructed using *J*(NO_2_) following the methods in [32,39]:

(8) $$\gamma_{g}=\;\;8.0\times 10^{-6}\times (1+\frac{J\left({NO}_{2}\right)}{{J\left({NO}_{2}\right)}_{noon}})$$

(9) $$\gamma_{a}=\;1.9\times 10^{-6}\times (1+\frac{J\left({NO}_{2}\right)}{{J\left({NO}_{2}\right)}_{noon}})$$

The above mentioned variation range of $\gamma_{g}$ and $\gamma_{a}$ and their influence on calculations results were evaluated in the sensitivity study.

Aside from the heterogeneous conversion of NO_2_, HONO can also be produced upon nitrate (${NO}_{3}^{-}$) aerosol photolysis ($P_{{NO}_{3}^{-}}$), which can be estimated using the observed ${NO}_{3}^{-}$ concentration and the photolysis rate of *J*(${NO}_{3}^{-}$):

(10) $$P_{{NO}_{3}^{-}}=\;J\left({NO}_{3}^{-}\right)\;\times \;\left[{NO}_{3}^{-}\right]$$

where *J*(${NO}_{3}^{-}$) was parameterized following previous work [71,72]:

(11) $$J\left({NO}_{3}^{-}\right)=\frac{8.3\;\times \;10^{-5}}{7\;\times \;10^{-7}}\;\times J({HNO}_{3})$$

(12) $$J\left({HNO}_{3}\right)=3.6\;\times \;10^{5}\;\times \;{J(HONO)}^{4}-7.3\;\times \;10^{2}\;\times \;{J(HONO)}^{3}+0.65\;\times \;{J(HONO)}^{2}-3.9\;\times \;10^{-5}\;\times J(HONO)-3.6\;\times \;10^{-10}$$

where *J*(HNO_3_) is the photolysis rate of gaseous HNO_3_. In the sensitivity study, a $J\left({NO}_{3}^{-}\right)$ bias of ±20% was considered.

The most important consumption pathway of HONO is its own photolysis decomposition process:

(R3) $$HONO+\;\;hv\overset{J\left(HONO\right)}{\to }\;NO+OH$$

with a HONO loss rate of:

(13) $$L_{hv}=J\left(HONO\right)\;\times \;\;\left[HONO\right]$$

Otherwise, HONO is mainly lost through dry deposition:

(14) $$L_{dep}=\frac{v_{d}}{PBLH}\;\times \;\;\left[HONO\right],$$

where v*_d_* is the dry deposition velocity of HONO, which is assumed to be 0.5 cm s^−1^ [19,73], PBLH is the planetary boundary layer height (obtained from ERA5 reanalysis data (https://cds.climate.copernicus.eu/#!/home, accessed date: 27 October 2023). By integrating the above sources and sinks, $P_{HONO}^{net}$ was calculated as:

(15) $${\;P}_{HONO}^{net}=P_{Vehicle}+P_{homo}+P_{ground}+P_{aerosol}+P_{{NO}_{3}^{-}}-L_{hv}-L_{dep},$$

and the unknown source/sink of HONO can be obtained using Equation (16):

(16) $$P_{unknown}=\frac{d\left[HONO\right]}{dt}-P_{HONO}^{net}$$

### 2.4. Derivation of Soil and Dew Water HONO Emissions

In agricultural regions, possible processes that could have been behind $P_{unknown}$ are mainly soil emissions and dew formation/evaporation (given there were no biomass burning events). In recent years, soil emissions of nitrogen containing gases have been widely studied in the fields of soil science, agriculture, and atmospheric science. As already mentioned in Section 2.3, soil emissions of HONO were determined using HONO flux measurements and parameterizations were established using its linear relationship with temperature, which only exists when emissions are present. However, there are commonly no or only very weak HONO emissions under temperatures below a certain threshold, where previous parameterizations would yield irrational negative emission rates. Additionally, the emission factor should vary with distinct soil conditions (soil water content and concentration of nitrogen species), which the typically used linear regression with fixed coefficients cannot reflect. Dew and guttation water formation and evaporation processes are highly complicated and their impact on HONO is even harder to parameterize.

In this study, we assume that all the $P_{unknown}$ was contributed by soil emissions and dew deposition/emissions. In previous field observations, dew water has mostly evaporated by noontime, only remaining until afternoon hours under strong daytime fog events. Thus, we adopted the time window of 13:00 to 19:00, during which we assumed that only soil emissions contributed to $P_{unknown}$, avoiding the time range when dew evaporation might overlap with soil emissions.

Thus, we could perform a fitting between *T_soil_* and $P_{unknown}$ within this time window for each day, to yield a parameterization for *P_soil_* that varies with each day. During the fitting process, we found that the exponential fitting equation (Equation (17)) could best describe the relationship between $T_{soil}$ and $P_{unknown}$.

(17) $$P_{soil}={A\;}_{soil}\;\times e^{B_{soil}\;\times \;T_{soil}}$$

$A_{soil}$ and $B_{soil}$ in Equation (16) are unknown constants that were determined during the fitting. Negative $A_{soil}$ and $B_{soil}$ were considered unreasonable, and the contribution of soil emissions to HONO was considered negligible on that day. Thus, ${P\left(\mathrm{HONO}\right)}_{soil}$ over the whole day could be derived by using the diurnal variation of $T_{soil}$. Using the exponential form to parameterize soil emissions instead of the traditional linear fitting approach could better reflect the fact that HONO emissions are close to zero beneath a certain temperature threshold. Additionally, parameters *A_soil_* and *B_soil_* varied from day to day, reflecting changes in soil emission strength impacted by environmental factors other than soil temperature.

By subtracting derived $P_{soil}$ from $P_{unknown}$, we obtained the remaining unknown HONO source/sink, which was attributed to dew emissions/depositions $P_{dew}$:

(18) $$P_{dew}=P_{unknown}-P_{soil}$$

with positive values representing HONO emissions during dew evaporation and negative ones representing HONO deposition due to its dissolution in dew water.

## 3. Results and Discussions

### 3.1. Variations of HONO and NH_3_

**Figure 1:**
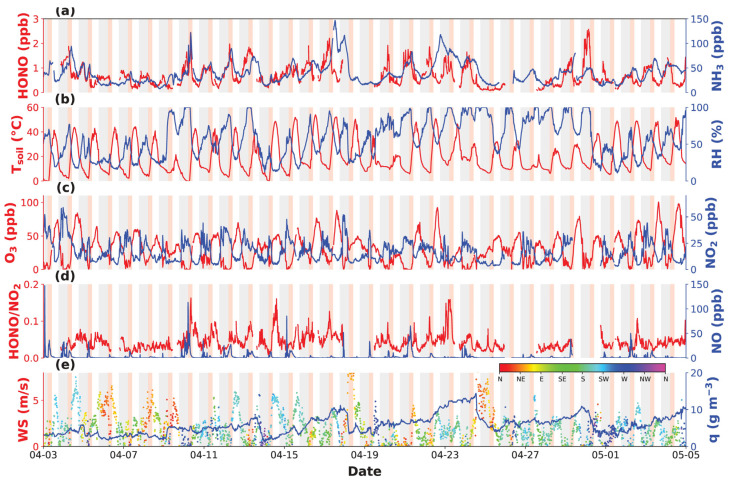
Measurements of (**a**) HONO and NH_3_, (**b**) soil temperature (*T_soil_*) and RH, (**c**) O_3_ and NO_2_, (**d**) HONO/NO_2_ and NO, (**e**) wind speed, direction (denoted by colors), and specific humidity (q) at GC Station in spring 2019. Grey shadings mark out nighttime periods (18:00 to 8:00) and red shadings denote periods with HONO morning peak occurrences.

**Figure 2:**
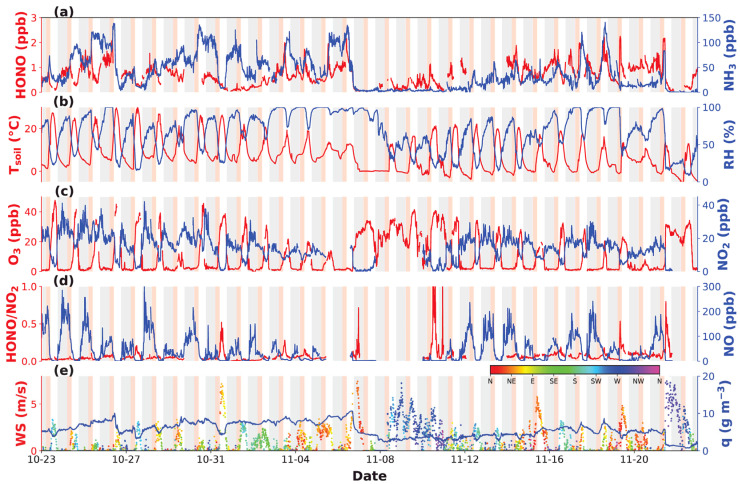
Measurements of (**a**) HONO and NH_3_, (**b**) soil temperature (*T_soil_*) and RH, (**c**) O_3_ and NO_2_, (**d**) HONO/NO_2_ and NO, (**e**) wind speed, direction (denoted by colors), and specific humidity (q) at GC Station in autumn 2021. Grey shadings mark out nighttime periods (18:00 to 8:00) and red shadings denote periods with HONO morning peak occurrences.

Figure 1 and Figure 2 present the time series of HONO, NH_3_, related gaseous pollutants and meteorological parameters measured during the spring of 2019 and the autumn of 2021 at GC, respectively. In spring 2019, HONO concentrations fluctuated between 0.08 and 2.6 ppbv, with an average of 0.7 ± 0.4 ppbv, while in autumn 2021, HONO concentrations fluctuated between 0.04 and 2.3 ppbv, with an average of 0.7 ± 0.4 ppbv. NH_3_ concentrations varied from 7.9 to 146.7 ppbv in spring, with an average of 40.2 ± 22.1 ppbv, while that in autumn ranged from 1.5 to 140.2 ppbv, with an average of 37.9 ± 33.1 ppbv. There was no significant difference in HONO concentrations between the two seasons, while NH_3_ concentrations in spring were only slightly higher than those in autumn, with a difference of ~2 ppbv.

The observed HONO and NH_3_ concentrations in both spring and autumn were similar to previously reported values for nearby urban and rural areas during the same seasons [20], but were generally higher than measurements in rural areas of European countries during the same seasons (Table 1). Compared to HONO observations in late autumn 2016 at GC, HONO reported in this study were much lower, while NH_3_ was significantly higher [48]. Another rural site very close to GC (Wangdu station, 70 km south of GC) is also representative of polluted rural NCP. Comparing observations in Wangdu and GC, a drastic drop in HONO levels was observed from 2016 to 2019 (Table 1), which might have been due to stringent air pollution control strategies implemented since 2013.

**Figure 3:**
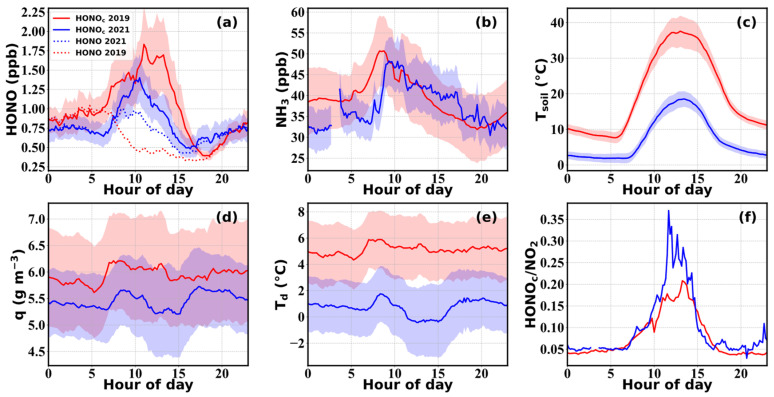
Diurnal variations in (**a**) HONO_c_ (solid line) and HONO (dashed line), (**b**) NH_3_, (**c**) *T_soil_*, (**d**) *q*, (**e**) dew temperature (*T_d_*), and (**f**) HONO_c_/NO_2_ observed in GC during spring 2019 (red) and autumn 2021 (blue).

This was supported by the fact that NO_x_ levels also significantly decreased from 2016 to 2021, with NO_x_ reaching averages of 80.6 ± 44.7 ppbv, 19.3 ± 14.6 ppbv and 57.4 ± 52.1 ppbv in autumn 2016, spring 2019 and autumn 2021, respectively. NO_2_ concentrations in autumn 2021 (14.2 ± 7.5 ppbv) were significantly lower than those in 2016 (34.7 ± 13.8 ppbv) and were also lower than those in the spring of 2019 (16.6 ± 9.8 ppbv). Since NO_x_ is typically high during cold seasons and low during warm seasons, this further supports the notion that NO_x_ reductions were prominent under stringent emission control measures. Although NO_2_ concentrations were lower in autumn 2021, the NO_2_ to HONO conversion rate (HONO/NO_2_) was higher than during spring 2019 (Figure 3f), indicating that environmental conditions in autumn 2019 might have been more favorable for heterogeneous NO_2_ to HONO conversions.

Figure 3 shows the average diurnal variations of observed HONO concentrations and those corrected for its photolysis (HONO_c_), as well as NH_3_ concentrations and other related parameters for spring 2019 and autumn 2021. A morning increase was detected in the averaged diel cycle of observed HONO during autumn 2021, but hardly for spring 2019. A common daytime decrease in uncorrected HONO concentration was noted, which was mainly caused by its strong photolysis loss that exceeded its production and emission rates. The daytime concentration of HONO in autumn was higher than that in spring, and remained at a relatively high level for a longer period of time. After the correction for its photolysis, HONO_c_ concentrations exhibited significant growth during the morning in both seasons, and concentrations were overall higher during spring than autumn, especially during noontime hours. After sunset, HONO concentrations started to accumulate throughout the night until reaching another peak on the next day. In autumn, a slight decrease was observed in HONO during the night before sunrise, which was not detected in spring. The average diurnal variation of HONO_c_ reached a daily maximum of 1.8 ± 0.5 ppbv around 11:00 LT and a minimum level of 0.4 ± 0.1 ppbv around 16:00 LT in spring. In autumn, the HONO_c_ reached its maximum (1.4 ± 0.3 ppbv) and minimum (0.5 ± 0.1 ppbv) around 10:00 LT and 15:00 LT, respectively; overall occurring an hour earlier than in spring, possibly due to differences in its budget between the two seasons. The differences in solar radiation conditions resulted in earlier onset, longer duration, and elevated strength in HONO photolysis loss during spring compared to that in autumn. Additionally, differences in atmospheric and soil temperatures and humidity between spring and autumn both impacted HONO soil emission strength, as well as the formation of dew and guttation water that determines the source and deposition strength of HONO within the aqueous phase.

The average diurnal variation of NH_3_ displayed a diel pattern similar to that of HONO_c_, with a gradual increase after sunrise. NH_3_ declined after sunset during autumn, which was inconsistent with HONO, while it increased after sunset during spring as HONO also did. While the peak time was similar during autumn, an earlier peak was observed in NH_3_ than in HONO_c_ during spring. It is also worth noting that the start time of the morning HONO increase was consistent with that of NH_3_ during both spring and autumn. The difference in NH_3_ peak concentration between spring and autumn was insignificant, with an average peak concentration of 50.7 ± 8.3 and 48.2 ± 5.7 ppbv in spring and autumn, respectively.

Among the 33 days of HONO observations in the spring, HONO morning peaks were observed 23 times (70%), while NH_3_ morning peaks were observed 22 times (67%), with simultaneous HONO and NH_3_ morning peaks (“dual-peak” phenomenon) occurring on 19 days (58%). No morning peaks were detected under rainy or foggy meteorological conditions. During autumn, HONO and NH_3_ morning increases were reported to occur almost on all days with valid observations. Note that specific humidity (q), which reflects the absolute amount of water vapor in air, also revealed morning increases simultaneous to those of HONO and NH_3_, indicating that dew water evaporation might be determining the HONO and NH_3_ morning growth, which was proved for the 2021 autumn campaign [39].

**Figure 4:**
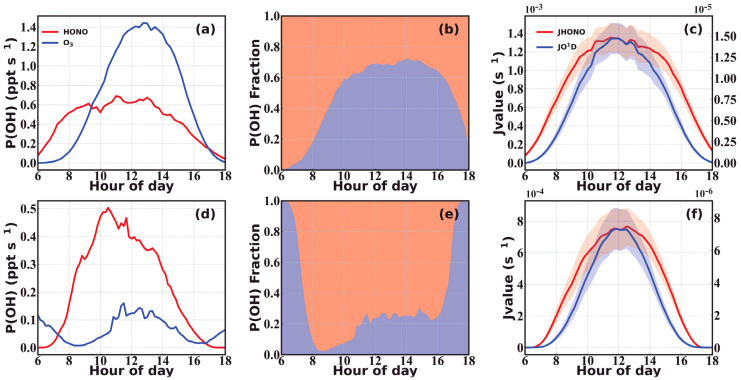
(**a**,**d**) Daytime OH production rates (P(OH)) of HONO and O_3_, (**b**,**e**) relative contributions of HONO and O_3_ to the total P(OH), and (**c**,**f**) photolysis rates of HONO and O_3_ during (**a**–**c**) spring and (**d**–**f**) autumn.

The continuous increase in HONO after sunrise indicated strong secondary formations or emissions that dominated over its deposition and photolysis loss during the morning, under relatively low solar radiation intensity and weak dry deposition. After sunrise, the persistent photolysis of the produced or emitted HONO would lead to rapid generation of OH radicals, increasing atmospheric oxidation capacities, initiating the oxidation of primary air pollutants and contributing to the formation of secondary gaseous pollutants (such as O_3_ or peroxyacetyl nitrate) and secondary aerosols (${NO}_{3}^{-}$, ${SO}_{4}^{2-}$ and secondary organic aerosol, SOA) [79]. Aside from HONO photolysis, OH radicals can also be produced during the photodissociation of O_3_. The absolute and relative contributions of both O_3_ and HONO to OH radical production rates (P(OH)) during spring and autumn are shown in Figure 4a,b,d,e, respectively. During the entire spring observation period, the daytime average relative contribution of HONO to the total P(OH) reached 40 ± 14%, dominating before 9:30 LT and after 17:00 LT (Figure 4b). The high springtime O_3_ concentration allows it to dominate the noontime P(OH), reaching a maximum of ~70%. Conversely, during the cooler autumn season with lower O_3_ concentrations, the contribution of HONO to the total OH production rate can reach a daytime average of 82 ± 9% and a maximum of 97% during the morning. Therefore, HONO played a deterministic role in OH production during autumn and despite the lower contributions of HONO in spring, it still played an important role in the generation of daytime OH radicals, especially during early morning hours, which may enhance the daytime formation of O_3_, thereby feeding back on OH production.

Our previous study demonstrated that the formation and evaporation of dew and guttation water had significant impacts on the HONO diurnal budget during autumn, storing atmospheric HONO within the aqueous phase in the form of nitrite over nighttime and releasing HONO during the daytime evaporation process [39]. The influence of such a process on HONO under springtime meteorological and environmental conditions, however, has not been investigated before. The formation of dew depends on environmental RH over the plant, soil, and ground surfaces. The amount of guttation, however, is mostly influenced by soil moisture and the amount of water transported from roots to leaf tips and edges by the plants after sunset. Compared to dew formation, which requires high RH conditions, guttation occurs far more frequently. In the morning, as the temperature rises, water drops on plant and soil surfaces evaporate, releasing water vapor and HONO, as well as other water-soluble trace gases such as NH_3_. RH conditions strongly influence the rate of water evaporation and thus also determine the rate and time of HONO emissions.

**Figure 5:**
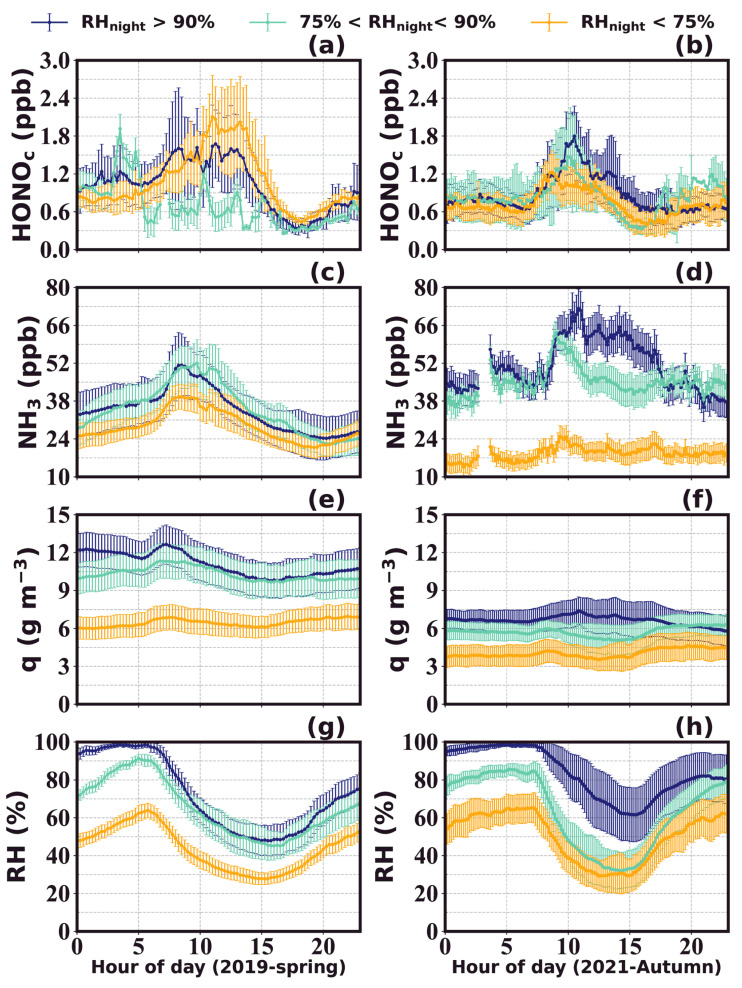
The average diel variations of HONO_c_ (**a**,**b**), NH_3_ (**c**,**d**) and *q* (**e**,**f**) in spring 2019 and autumn 2021 under different relative humidity conditions (**g**,**h**). Dark blue for high relative humidity conditions (RH_night_ > 90%), pale green for medium relative humidity conditions (75% < RH_night_ ≤ 90%), orange for low relative humidity conditions (RH_night_ < 75%).

In the 35-day observation period during spring, there were 17 days with nighttime RH exceeding 75%, accounting for approximately 50% of the total number of days. Among those days, there were 11 days with nighttime RH exceeding 90%. In the 33-day observation period in autumn, there were 21 days with nighttime RH exceeding 75%, accounting for more than 60% of the total number of days. Among these, there were 12 days with nighttime RH exceeding 90%. High RH conditions provided favorable conditions for the nighttime formation of dew, whose evaporation led to increases of q during daytime (Figure 5e,f). Additionally, the longer duration of dew and fog droplet evaporation, under stable atmospheric conditions, can maintain the presence of liquid water and dew until mid-afternoon, leading to an extended period of water vapor growth, as shown in Figure 5e,f. Under stable meteorological conditions, the concentrations of HONO_c_ and NH_3_ in the atmosphere remained relatively stable before sunrise. Afterwards, during the rapid evaporation of water droplets, both HONO_c_ and NH_3_ experienced rapid growths lasting for approximately 1–2 h (Figure 5a,b,e,f). The average growth rates of HONO_c_ and NH_3_ during this stage reached 0.5 ± 0.2 and 22.0 ± 8.1 ppb h^−1^ in spring, and 0.8 ± 0.3 and 20.0 ± 15.2 ppb h^−1^ in autumn, respectively. Under higher RH conditions, the daily average increments of HONO_c_ and NH_3_ were significantly higher than those observed under lower RH conditions. However, in terms of relative increments, there were no significant differences between high and low RH conditions. During springtime, the HONO_c_ diurnal profile under high RH conditions revealed two separate peaks, which suggests that there might have been two distinct HONO production or emission pathways leading to these two peaks. Prenoon HONO_c_ was the highest on days following nighttime high RH conditions, suggesting that the prenoon HONO production or emission process was promoted by humid conditions. However, noontime HONO_c_ was the highest following low RH nights, which suggests that low RH conditions were most favorable for the noontime HONO production or emission. The prenoon source of HONO corresponds well with previously confirmed daytime dew and guttation evaporation and emissions, while the noontime source may be dominated by soil emissions, which were probably enhanced on clear and dry days when the fertilized soils were sufficiently heated by solar radiation. During autumn, prenoon rises in HONO_c_ were observed under all nighttime RH conditions, with the peak occurring earlier under low nighttime RH and later under higher nighttime RH. The highest HONO_c_ peak was observed following high RH nighttime conditions. This may be attributed to weak HONO soil emissions during the cold late autumn season, which rendered dew evaporations (that occurred earlier during the day when RH was lower) to be the dominant contributor to HONO_c_ variations. To further elucidate how much of the observed HONO could not be explained by currently known and accounted sources during the spring and autumn seasons, we performed a detailed budget study that is presented in the following section.

### 3.2. Budget of HONO

**Figure 6:**
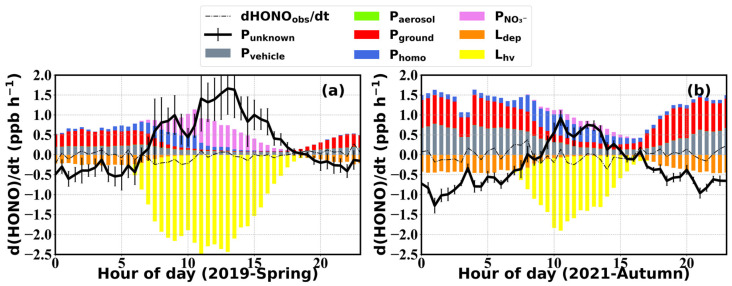
Averaged diurnal variation in HONO budgets during (**a**) spring 2019 and (**b**) autumn 2021. Gray, green, red and pink bars represent vehicle HONO emissions, heterogeneous transformation of NO_2_ to HONO on aerosol and ground surfaces, and HONO production during nitrate photolysis, respectively, while orange and yellow bars show dry deposition and photolysis removal of HONO.

The contribution of various HONO sources and sinks to the overall HONO budget during spring and autumn are displayed in Figure 6, with the black line representing the unknown HONO production/destruction rate (*P_unknown_*) that could not be explained with processes currently accounted for. *P_unknown_* was significantly higher during spring 2019 than in autumn 2021, exhibiting two daytime peaks (around 9:00 and 14:00) in spring and only one peak (from 10:00 to 14:00) in autumn.

**Figure 7:**
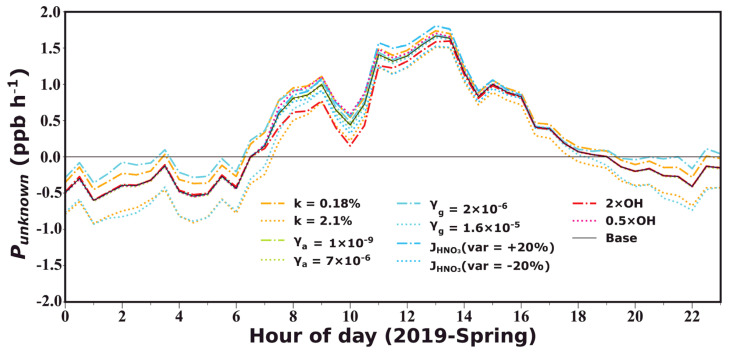
The unknown variation rate of HONO (*P_unknown_*) under different parameter assumptions. The solid black line represents the base case. The orange dashed and dotted lines represent *P_unknown_* calculated using *k* = 0.18% and 2.1% as the lower and upper limits of vehicle emission coefficients obtained in previous tunnel experiments [13,56], respectively. The green dashed and dotted lines represent calculation results adopting *γ_a_* = 1 × 10^−9^ and 7 × 10^−6^ as the lower and upper limits of aerosol surface uptake coefficients according to laboratory measurements summarized in Li, Su, Li, Ma, Pöschl and Cheng [67], respectively. The turquoise dashed and dotted lines represent those applying *γ_g_* = 2 × 10^−6^ and 1.6 × 10^−5^ as the lower and upper estimates for ground surface uptake coefficients of NO_2_ following the field measurement results of VandenBoer, et al. [66], respectively. The blue dashed and dotted lines represent the results obtained by adjusting the nitrate photolysis coefficient down or up by 20%, respectively. The red dashed and dotted lines represent the results obtained by multiplying [OH] by 2 and 0.5, respectively.

The estimated vehicle emissions revealed similar diurnal variation patterns in spring and autumn, with the contribution being higher during nighttime and decreasing after sunrise to a minimum in the afternoon, following the diel cycle of NO_x_. The averaged contribution in autumn (0.44 ± 0.42 ppb h^−1^) was higher than that in spring (0.15 ± 0.12) due to elevated cold season NO_x_ concentrations. Uncertainties in the HONO/NO_x_ emission ratio introduced averaged deviations of −0.24 ± 0.09 to 0.11 ± 0.04 ppb h^−1^, with larger deviations during nighttime (20:00–8:00 LT, deviations between −0.31 ± 0.05 and 0.14 ± 0.02) than daytime (8:00–20:00 LT, deviations between −0.17 ± 0.05 and 0.08±0.02 ppb h^−1^). Overall, uncertainties in vehicle emission parameterization were among the most important contributors to *P_unknown_* estimation errors; however, they exerted insignificant influences on the daytime *P_unknown_* and the derivation of dew and soil HONO emissions (Figure 7). Note that the upper limit in the vehicle emission factor caused a larger deviation, which is however unrealistic, since such a high emission factor was scarcely reported. Additionally, the assumption that all observed NO_x_ came from vehicle emissions was already responsible for an overestimation in *P_vehicle_*, implying that the actual *P_vehicle_* might be far lower than that estimated using the upper limit of *k* = 2.1%.

*P_homo_* mostly contributed to prenoon *P_unknown_* during spring, while its peak shifted to later hours during autumn. Larger contributions to the HONO budget were observed during autumn (0.20 ± 0.41 ppb h^−1^) rather than in spring (0.09 ± 0.24 ppb h^−1^), also due to higher cold season NO levels. Estimation uncertainties in *P_homo_* mainly come from OH radical concentrations parameterizations. Using parameterization schemes proposed based on measurements in Wangdu during summer and winter seasons, we yield OH concentrations within a reasonable variation range (<1.3 × 10^7^ and 3.1 × 10^6^ molec m^−3^ during spring and autumn, respectively). Assuming a 20% uncertainty in [OH] estimates, *P_unknown_* fluctuated on average within −0.17 ± 0.10 to 0.08 ± 0.05 ppb h^−1^ during daytime prenoon hours, while during the rest of the day its influence was negligible (−0.01 ± 0.03 to 0.003 ± 0.01 ppb h^−1^).

*P_ground_* also made relatively higher contributions to HONO in autumn (0.49 ± 0.49 ppb h^−1^) compared to spring (0.23 ± 0.38 ppb h^−1^), which is also reflected in the higher HONO_c_/NO_2_ ratios in Figure 3f. *P_ground_* mostly contributed to nighttime *P_unknown_* due to elevated S/V, despite photo-enhanced NO_2_ uptake assumptions that increased *γ_g_* by an order of magnitude [69,70,71], exceeding contributions of *P_vehicle_* during nighttime. In comparison, NO_2_ conversions on aerosol surfaces were negligible in both seasons (0.003 ± 0.003 ppb h^−1^). Thus, uncertainties in NO_2_ uptake coefficients were more important for the accuracy of *P_unknown_* estimations, especially during nighttime. The upper limit in *γ_g_* resulted in averaged *P_unknown_* deviations of −0.18 ± 0.14 ppb h^−1^, with nighttime deviations similar to those resulting from the upper limit of the vehicle emission coefficient. The lower limit in *γ_g_* resulted in 0.13 ± 0.10 ppb h^−1^ deviations, which exceeded the deviations introduced by the lower limit of the vehicle emission coefficient. It should be noted that NO_2_ uptake might be enhanced on wet surfaces, such as dew water, moist soil, wet aerosols, and fog water, however, there is currently no appropriate parameterization to describe such behavior. Thus, there might be overestimations in the nighttime *P_unknown_*. Since we mostly focus on daytime *P_unknown_* in the later section, this might not be of specific concern.

HONO formation from the photolysis of nitrate was higher during spring (0.17 ± 0.33 ppb h^−1^) and much lower in autumn (0.04 ± 0.06 ppb h^−1^), on the one hand, due to stronger solar radiation during spring and on the other hand due to decreases in nitrate mass concentrations from 2019 to 2021. Uncertainties in nitrate photolysis rate estimations of 20% brought on deviations in *P_unknown_* of ±0.12 ppb h^−1^ between 8:00 and 14:00, while during the rest of the day influences were very small.

Regardless of spring or autumn, photolysis was the main sink for HONO during the day, exerting a strong effect on daytime HONO, especially between 7:00 and 18:00 LT in spring and between 9:00 and 15:00 in autumn, during which the photolysis loss rate exceeded the sum of the aforementioned sources.

The unknown variation rate of HONO, *P_unknown_*, exhibited a clear diel variation with negative values during nighttime and strong positive ones during daytime, especially towards noontime. Daytime *P_unknown_* exceeded the HONO production rates of all accounted sources during noontime hours in spring and reached comparable levels to the total accounted P_HONO_ in autumn. Nighttime *P_unknown_* was far greater than the deposition loss of HONO in both spring and autumn. This further emphasized the urgency and importance of unraveling these unknown sources for a better representation of HONO formation in atmospheric chemistry-related observational and modelling studies.

Overall, uncertainties in nighttime *P_unknown_* are greater than those during the daytime, which were mostly contributed by coefficients used in the calculation of vehicle emissions and NO_2_ conversion on the ground surface. Daytime uncertainties were mostly attributed to [OH] estimation and nitrate photolysis rate errors. Despite the existence of various uncertainties, *P_unknown_* was always maintained at high levels (with the average diurnal maximum ranging from 1.57 to 1.83 ppb h^−1^), indicating that missing sources of HONO indeed existed and that the discrepancy was not caused by uncertainties within the HONO budget calculation.

To maintain such high HONO concentrations at noontime, there must have been strong HONO emission sources or production pathways during the day with emission intensities of up to 7 ppb h^−1^, in addition to the sources that were already accounted for in the budget analysis. Microorganisms in soil widely exist that can produce NO_2_^−^ as a byproduct during nitrification processes. Soil emissions of HONO were suggested to be especially pronounced within farmlands with both extensive vegetation coverage and large loads of fertilizer application [63,80], and would be promoted by higher atmospheric temperatures and lower RH, thus exhibiting noontime peaks. Aside from possible contributions from soil emissions, our previous study suggested that there were significant emissions from dew and guttation water during autumn, with rapid emissions within relatively short time windows mostly during prenoon hours. However, under extremely stable foggy conditions, the emission process can be extended until early afternoon hours [39]. Earlier studies have also suggested that HONO was also generated through liquid-phase chemical reactions aside from simple dissolution loss and evaporative emissions.

In spring, *P_unknown_* peaked around 9:00 and 13:30 LT, and was observed on particular days, possibly related to dew evaporation and soil emissions. In autumn, the average *P_unknown_* increased during the day and displayed small peaks between 10:30 and 13:30. It should be noted that the averaged result does not necessarily reflect the true diel variations of *P_unknown_*, especially during autumn, when peaks occurred during distinct hours of the day (from 11:00 to 13:00). This might have been due to the aforementioned distinct time windows of dew HONO emissions depending on atmospheric stability and evaporation conditions. Negative nighttime *P_unknown_* suggests that there were unaccounted sinks of HONO, which correspond well to its dissolution in near-surface liquid water, that were not considered in the budget study. This deposition loss was stronger during spring than autumn, possibly due to the dense coverage of grown wheat leaves and larger amounts of dew and guttation water. The daytime unknown source of HONO might have been commonly contributed by dew water evaporation and soil emissions, which have resulted in elevated *P_unknown_* during distinct time windows. Based on these premises, we try to separate these two emission sources and quantify their source strength in the next section.

### 3.3. Soil Emissions of HONO and NH_3_

Using methods described in Section 2.3, diel variations of soil emissions (*P_soil_*) were fitted using an afternoon time window, which was believed to be undisturbed by dew and guttation water emissions. Afterwards, evaporative emissions from dew water (*P_dew_*) were deduced by subtracting soil emissions from *P_unknown_*.

**Figure 8:**
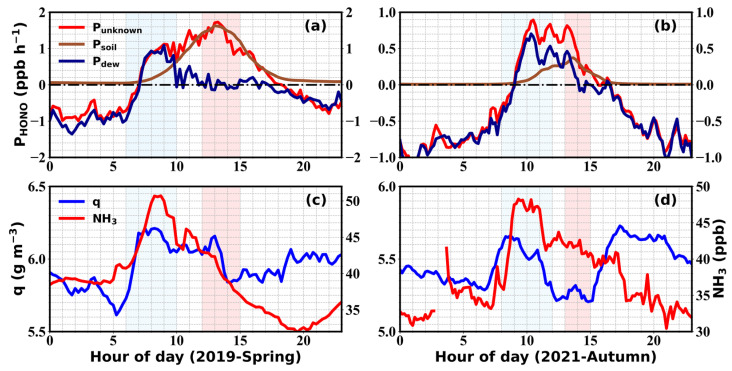
Diel profiles of *P_unknown_* (red line) with calculated soil (brown line) and dew (dark blue line) HONO emissions during (**a**) spring and (**b**) autumn, as well as the corresponding changes in *q* and NH_3_ during (**c**) spring and (**d**) autumn. The light blue shading denotes the time range from 6:00 to 10:00 LT, corresponding to the peak of *P_dew_*, while the red shading denotes the time range from 12:00 to 15:00 LT, corresponding to the peak of *P_soil_*.

From the springtime fitting results, *P_unknown_* and *P_dew_* both increased after sunrise and reached a first peak of 1.12 ± 1.13 ppb h^−1^ at around 9:00 LT on average (Figure 8a), which was in high accordance with the drastic increase and peak in water vapor and NH_3_ (Figure 8c). Soil emission remained insignificant during this period due to relatively lower soil temperatures, however, with the increase in temperature after 9:00 LT, *P_soil_* increased and *P_dew_* drastically decreased under the rapid evaporation of dew drops and remained near zero until guttation started again (near sunset). As the soil temperature rose and reached its average peak at 13:00 LT, *P_soil_* also reached its highest value of 1.63 ± 1.37 ppb h^−1^ throughout the day, which compensated for the missing noontime source. Before 7:00 and after 17:00, *P_unknown_* was dominated by the negative contribution of *P_dew_*, whose role as a sink for HONO was not considered in the budget study. Altogether, springtime soil emissions impacted HONO throughout the day, with a broad peak and a maximum during noontime hours, while dew emissions were only present during prenoon hours before their complete evaporation, resulting in drastic morning time HONO emissions within relatively short time scales. Integrating over the entire day, *P_soil_* generally contributed more strongly to daytime HONO than *P_dew_* during springtime, both due to large loadings of nitrogen-containing fertilizer application and more favorable meteorological conditions (higher temperatures, stronger solar radiation, and lower RH) for soil emissions. During the spring observations, only on 7 April, dew emissions were comparable to soil emissions throughout the entire daytime period, which was due to minimal temperature increments and thus lower soil temperatures, as well as an extended dew evaporation and emission window on that day. Additionally, it should be noted that NH_3_ could also be emitted from fertilized soils, however, soil emissions had less impact on the overall diurnal variation of NH_3_ compared to dew emissions (Figure 8c).

During autumn, *P_dew_* increased after 8:30 LT and reached a several peaks between 10:00 and 12:00 LT, whose peak heights (0.56 ± 1.13 to 0.90 ± 0.96 ppb h^−1^) were higher than that of *P_soil_* (0.40 ± 0.89 ppb h^−1^) reached near 13:30 LT (Figure 8b). The average peak time corresponded well with those of *q* and NH_3_ (Figure 8d), with dew emissions also having more pronounced impacts than soil emissions on NH_3_ during autumn. Compared to the spring season, the autumn season displayed lower temperatures, leading to a ~1-h delay of the dew emission window. *T_soil_* also rose more slowly and exhibited a noticeably lower peak compared to the spring. As a result, there was an overlap in the emission windows of dew and soil emissions during noontime hours (11:00 to 13:00 LT) in autumn. Compared to spring, *P_dew_* turned negative earlier during the afternoon and stayed negative until later hours in the morning during autumn due to earlier sunsets and later sunrises. Frequently occurring stagnant atmospheric conditions favored the formation and prolonged the lifetime of fogs, dewfall, and plant guttation in autumn, which all acted as sinks for atmospheric HONO.

**Figure 9:**
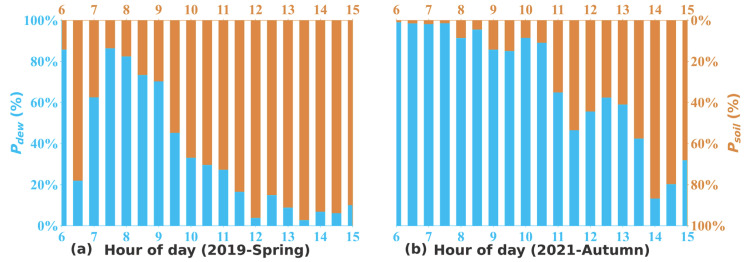
The relative contribution of *P_dew_* (blue) and *P_soil_* (brown) to $P_{unknown}$.

The diurnal variations of the relative contribution of *P_dew_* and *P_soi__l_* to *P_unknown_* during spring and autumn are depicted in Figure 9. During spring, dew evaporation dominantly contributed to the unknown source of HONO from 6:00 to 9:00 LT in the morning, with contributions decreasing gradually from 88% to 70%. Soil emissions took over as dew completely evaporated and became dominant after 10:00 LT, with an average relative contribution of 88% from 10:00 to 15:00 LT. In autumn, dew emissions predominantly contributed to *P_unknown_* before 13:00, with relative contributions decreasing from 99% to 54% during the period from 6:00 to 13:30, which could be attributed to weaker radiative heating and thus lower air and soil temperatures that resulted in both weaker soil emissions and slower evaporations of dew and guttation water (Figure 8b). Therefore, it could be concluded that the sudden increases in HONO concentration and *P_unknown_* during the morning were positively correlated to dew water evaporation as denoted by simultaneous increments in q (Figure 8d), while the high afternoon levels were largely maintained by soil emissions. Due to the alternating contributions of these two sources throughout the day, the averaged HONO diurnal variation typically exhibited overlayered morning and afternoon peaks during daytime in the spring and autumn seasons.

Overall, it can be concluded that dew and soil emissions both had important impacts on the diel variations of HONO at GC, with dew emissions playing a major role before the afternoon in autumn and during early morning hours in the spring season. Soil emissions were more pronounced and recognizable during the spring season due to elevated temperatures and solar radiation, while those in autumn only contributed to HONO emissions within a short time window in the afternoon hours.

## 4. Conclusions and Implications

During the two observation periods, the concentration of HONO and NH_3_ was notably higher than those observed in urban areas, indicating the importance of rural areas to the emissions and background levels of HONO and NH_3_. This study provides a comprehensive analysis of the variations in HONO and NH_3_ during the spring and autumn seasons in rural areas of the NCP and explores the potential contributions of dew and soil emissions to their significant diurnal variations.

The large unknown daytime source and nighttime sink of HONO were manifested based on observations and theoretical calculations, with the unknown daytime source of HONO displaying distinct diel variations during spring and autumn. The results in this study revealed dew evaporation to be a significant process contributing to the morning peaks of unknown HONO sources, while soil emissions were the key drivers of high afternoon HONO levels. HONO is typically commonly deposited and accumulated in dew and liquid water droplets after sunset, reducing their dispersion in the atmosphere and resulting in their release in the morning during plant surface dew evaporation. After sunrise, rising soil temperatures also lead to the increase in soil emissions of evaporative trace gases including HONO.

The unknown daytime source and nighttime sink of HONO were both larger during spring than in autumn. Thus, the absolute contribution of dew and soil HONO emissions was also higher in spring. However, the relative contribution of dew emissions was higher during autumn, despite larger amounts of springtime dew water, which was mainly due to higher springtime soil temperatures. Nevertheless, dew emissios remained the dominant contributor to morning time HONO emissions in spring.

After sunrise, HONO is easily photolyzed and generates a substantial amount of OH radicals, initiating photochemical reactions leading to the formation of secondary air pollutants. During spring, HONO photolysis is the major contributor to OH production in the morning and during the late afternoon, while noontime contributions of 30~40% were also nonnegligible. During autumn, HONO photolysis contributed over 70% to OH production throughout sunlight hours, with morning contributions reaching over 90%. Thus, dew processes were determinative of atmospheric oxidation capacity during the morning and were responsible for the initiation of daytime photochemistry, while soil emissions further maintained P(OH)_HONO_ at a high level, especially during spring.

The formation and evaporation of liquid water on plant surfaces is a frequently occurring process not only in agricultural ecosystems, but also in other vegetated environments, thus exerting far-reaching impacts on HONO over a large spatial scale. The consequences of these processes on atmospheric oxidizing capacity and the formation of secondary air pollutants have not been adequately recognized and emphasized in previous observational and modelling studies. This has led to a significant underestimation of daytime OH radical levels in the NCP, profoundly affecting the progression of daytime atmospheric chemical reactions and potentially causing significant discrepancies in secondary air pollution predictions. Future studies need to thoroughly investigate the influencing factors of dew and soil emissions and establish their relations to HONO emission rates, in order to form parameterizations for regional and global models.

## Figures and Tables

**Table 1:** Comparison between ground-based atmospheric HONO measurements at rural or suburban sites.

Location	Period	HONO (ppbv)	Reference
Paris/France	July 2009	0.01–0.50	[74]
Melpitz/Germany	April 2018	0.25 ± 0.11	[43]
Utah/America	January–February 2012	0–0.27	[75]
Seoul/Korea	June 2004	0.6 ± 0.8	[76]
Dongying/China	June–July 2017	0.17 ± 0.20	[77]
GC/China	October 2016	6.3 ± 4.6	[48]
Wangdu/China	June 2017	1.1 ± 1.2	[78]
Wangdu/China	December 2017	1.8 ± 1.4	[20]
GC/China	April 2019	0.7 ± 0.4	This study
Wangdu/China	June–August 2020	0.7 ± 0.5	[20]
Wangdu/China	September 2020	0.8 ± 0.6	[20]
GC/China	November 2021	0.7 ± 0.4	This study

## Data Availability

Data available on request from the corresponding author.
